# Extrasynaptic GABA_A_ Receptors and Tonic Inhibition in Rat Auditory Thalamus

**DOI:** 10.1371/journal.pone.0016508

**Published:** 2011-01-26

**Authors:** Ben D. Richardson, Lynne L. Ling, Victor V. Uteshev, Donald M. Caspary

**Affiliations:** Department of Pharmacology, Southern Illinois University - School of Medicine, Springfield, Illinois, United States of America; Dalhousie University, Canada

## Abstract

**Background:**

Neural inhibition plays an important role in auditory processing and attentional gating. Extrasynaptic GABA_A_ receptors (GABA_A_R), containing α_4_and δ GABA_A_R subunits, are thought to be activated by GABA spillover outside of the synapse following release resulting in a tonic inhibitory Cl^−^ current which could account for up to 90% of total inhibition in visual and somatosensory thalamus. However, the presence of this unique type of inhibition has not been identified in auditory thalamus.

**Methodology/Principal Findings:**

The present study used gaboxadol, a partially selective potent agonist for δ-subunit containing GABA_A_ receptor constructs to elucidate the presence of extrasynaptic GABA_A_Rs using both a quantitative receptor binding assay and patch-clamp electrophysiology in thalamic brain slices. Intense [^3^H]gaboxadol binding was found to be localized to the MGB while whole cell recordings from MGB neurons in the presence of gaboxadol demonstrated the expression of δ-subunit containing GABA_A_Rs capable of mediating a tonic inhibitory Cl^−^ current.

**Conclusions/Significance:**

Potent tonic inhibitory GABA_A_R responses mediated by extrasynaptic receptors may be important in understanding how acoustic information is processed by auditory thalamic neurons as it ascends to auditory cortex. In addition to affecting cellular behavior and possibly neurotransmission, functional extrasynaptic δ-subunit containing GABA_A_Rs may represent a novel pharmacological target for the treatment of auditory pathologies including temporal processing disorders or tinnitus.

## Introduction

The medial geniculate body (MGB) is the thalamic nucleus of the central auditory system serving to shape and/or gate information as it is passed on to auditory cortical neurons. Like other sensory thalamic structures, the MGB is considered more than a simple relay nucleus as evidenced by recent data showing important roles for MGB neurons in coding stimulus specific adaptation and processing temporally complex stimuli [1], [2], [3]. The primary divisions of the rat MGB are the dorsal (MGd), medial (MGm) and ventral (MGv) [4], [5], [6]. The extralemniscal MGd and MGm have diverse afferents from the inferior colliculus (IC), auditory cortex (AC) and spinothalamic tract and efferents to the striatum, amygdala and areas of AC [7], [8]. The lemniscal MGv receives glutamatergic input from the IC and projects to the auditory cortex [7], [8]. In the rat MGB, the two major sources of inhibition are the GABAergic projections from IC and the thalamic reticular nucleus (TRN) as GABAergic interneurons compose <1% of the cellular population [7], [9], [10], [11], [12], [13]. As a result, TRN and IC inhibitory inputs likely shape MGB response properties through tonotopically or focused projections onto MGB neurons [1], [10], [14], [15], [16].

The GABA_A_ receptor (GABA_A_R) is a heteromeric member of the cys-loop superfamily. It forms a Cl^−^ permeable ion channel pore and serves as the primary inhibitory neurotransmitter receptor in the brain. Nineteen GABA_A_R subunits (α_1–6_, β_1–3_, γ_1–3_, δ, ε, θ, π and ρ_1–3_) are known, specific combinations of which form functional GABA_A_Rs. Extensively reviewed by others, GABA_A_Rs lacking the γ subunit and containing the δ-subunit (δ-GABA_A_Rs) are benzodiazepine insensitive, located extrasynaptically, show high ligand affinity, exhibit relatively slow desensitization and mediate a tonic inhibitory Cl^−^ current [17], [18]. Functional δ-GABA_A_Rs that mediate tonic inhibition and alter neuronal excitability are expressed in visual and somatosensory thalamocortical neurons, the dorsal lateral geniculate nucleus and ventrobasal complex, respectively [19], [20], [21], [22], but have not been reported in the MGB.

In thalamic nuclei, the incorporation of both the α_4_ and δ subunits within the same GABA_A_R construct appears required for tonic current activation and existing data indicate that these subunits preferentially co-assemble [20], [23], [24]. Survey studies reveal the presence of α_4_δ subunit mRNA in the MGB, suggesting that functional α_4_δ-GABA_A_R constructs could be present in rat auditory thalamus [25]. Collectively, these data underpin the rationale for the present set of experiments to identify the presence of functional α_4_δ-GABA_A_Rs and tonic inhibition in the MGB.

The present study used gaboxadol (formerly THIP), a δ-subunit specific agonist, which, when present at low (µM) concentrations, preferentially binds and activates non-γ_2_, δ-subunit containing GABA_A_Rs [17], [26] to both label and dose-dependently activate these receptor subtypes in auditory thalamocortical neurons.

## Materials and Methods

All experiments were completed using Fischer Brown Norway (FBN) male rats maintained on an ad libitum diet and reversed light-dark cycle. Procedures were in accordance to protocols approved by the Laboratory Animal Care and Use Committee of Southern Illinois University-School of Medicine (SIU Animal Protocol Numbers: 41-06-024 and 41-01-002).

### Quantitative Receptor Binding Autoradiography

FBN rats (11-months-old) were decapitated and brains were rapidly removed, rinsed in ice-cold phosphate buffer at 4°C (pH 7.4), frozen in powdered dry ice and stored at −80°C. Serial transverse sections were cut at 16 µm using a Leica CM1850 cryostat at −18°C. Selected sections were thaw-mounted onto Superfrost/Plus slides and stored at −20°C. Anatomical locations of the MGB were verified to match neural structures with those previously described [4].

[^3^H]Gaboxadol (Merck & Co. Inc., Rahway, NJ) was used with modified protocols from Milbrandt and Caspary [27] and Bjarke Ebert (personal communication). In brief, tissue sections were subjected to pre-wash twice for 5 minutes in buffers, followed by incubating with [^3^H]gaboxadol: 10–400 nM and post-wash with buffers for 4 quick dips. Buffer solutions used were 50 mM Tris-citrate (pH 7.1). Non-specific binding was determined in adjacent sections by the addition of cold excessive GABA to the ligand buffer.

Dried slides were apposed to [^3^H]-hypersensitive phosphor screens for 3–5 days at room temperature. The phosphor screens were scanned using a Cyclone storage phosphor system. The MGB was outlined and analyzed using OptiQuant image analysis software which provided tools for gray-scale quantification in digital light units (DLU). DLUs were then converted to nCi/mg protein using a standard curve generated from co-exposed ^3^H-embedded plastic standards (ARC, St. Louis, MO) [28].

### Voltage Clamp Whole Cell Recordings

22-30-day-old and 6-7-month-old FBN rats were anesthetized with 2.5–3.0% Isolfurane gas and decapitated. Brains were rapidly removed and placed in ice-cold solution containing (in mM): 250 sucrose, 2.5 KCl, 26 NaHCO_3_, 1.26 NaH_2_PO_4_, 5 MgCl_2_, 0.5 CaCl_2_, 10 glucose. To increase cell survivability, slices from six month old animals were collected in an identical sucrose based solution which also contained 2 mM kynurenic acid. Horizontal slices, 200–300 µm thick containing the ventral division of MGB were prepared using a Vibratrome 1000 Plus (Leica Microsystems GmbH, Wetzlar, Germany) and transferred to a storage chamber where slices were perfused for 30 minutes at 30°C with artificial cerebrospinal fluid (ACSF) containing (in mM): 125 NaCl, 3 KCl, 1.26 NaH_2_PO_4_, 2 CaCl_2_, 1 MgCl_2_, 26 NaHCO_3_, 10 glucose. Slices were then transferred to the recording chamber one at a time as needed. All recordings were conducted at room temperature.

Voltage-clamp recordings were conducted using a MultiClamp-700B amplifier and digitized by a Digidata 1440A (Molecular Devices, Sunnyvale, CA) at 5–10 kHz and filtered at 2–2.2 kHz. Data were analyzed offline with Clampfit 10.2. Gaboxadol (THIP), gabazine (SR-95531) and kynurenic acid were obtained from Sigma Aldrich (St. Louis, MO). Tetrodotoxin (TTX) was purchased from Tocris Biosciences (Ellisville, MO).

Patch-clamp recording pipettes were pulled from single-filament borosilicate glass (O.D. 1.5 mm, I.D. 0.86 mm) using a Sutter P-87 micropipette puller and were filled with an intracellular solution containing (in mM): 130 CsCH_3_SO_3_, 10 HEPES, 6 NaCl, 2 MgCl, 2 MgATP and 0.3 NaGTP with a pH of 7.33 adjusted with CsOH. The recording pipette tip resistance was 3–7 MΩ. Pipettes used for focal drug application were similar to the recording pipettes except for the tip resistance which was increased to 6–9 MΩ. Once a gigaseal (>1 GΩ seal) was obtained, the cell membrane was ruptured resulting in whole-cell access. Patches that exhibited a series resistance higher than 30 MΩ were improved by application of additional negative pressure or discarded. Voltage-clamp recordings were conducted at a holding voltage of −10 mV.

All experiments were conducted in ACSF containing 2–3 mM kynurenic acid to block ionotropic glutamate receptors. Gaboxadol was applied to the ACSF and gabazine, a selective GABA_A_R antagonist, was pressure-applied focally via a picospritzer pipette (1–3 psi) positioned 20–30 µm from the recorded cell. TTX (0.15 µM) was applied to ACSFto block voltage-gated Na^+^ channels in experiments using 22-30-day-old animals only.

## Results

### Receptor binding assay indicates high levels of [^3^H]gaboxadol binding in the MGB

**Figure 1:**
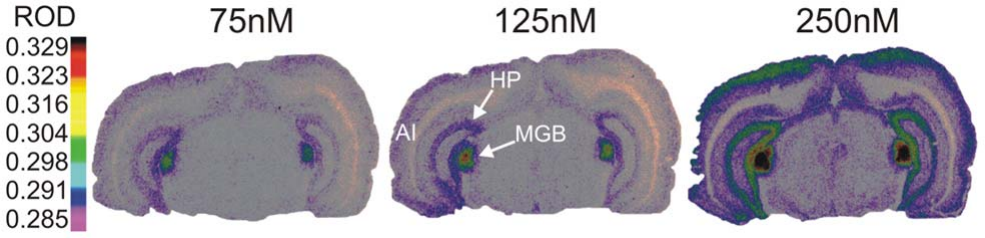
Receptor Binding Assay Indicating High Levels of δ-containing GABA_A_Rs on MGB Neurons: Representative autoradiographs of [^3^H] gaboxadol binding in young adult rats. Warm colors (red) indicate higher levels of binding while cooler colors (blue) represent lower levels (referenced to Relative Optical Density spectrum at left). At all three concentrations shown here (75 nM, 125 nM and 250 nM), [^3^H]gaboxadol binds selectively to GABA_A_Rs in MGB with little binding in brain regions shown in this coronal section, except for hippocampus and upper layers of neocortex. The MGB and hippocampus are indicated by arrows labeled “MGB” and “HP”, respectively with primary auditory cortex labeled as “A1”.

[^3^H]gaboxadol displayed high levels of binding at low ligand concentrations in the rat MGB (n = 4) (Figure 1). Saturation analysis indicates a B_max_ of 151.11±21.47 nCi/mg protein and K_d_ of 194.64±36.92 nM in the MGB (mean ± SEM). Figure 1 shows representative coronal sections through the MGB, displaying lower levels of [^3^H]gaboxadol binding in hippocampus. It is likely that other sensory thalamic areas, cortex, cerebellum, etc. known to express δ-GABA_A_R s would show similar levels of binding, but are not present in Figure 1 [29]. Regionally specific high levels of [^3^H]gaboxadol binding was seen in the MGB. This was in contrast to the relative absence of binding in neighboring structures, but consistent reports providing evidence for α_4_δ-GABA_A_Rs in the hippocampus [30], [31]. [^3^H]gaboxadol binding in both MGB and hippocampus contrasts with the relative absence of [^3^H]gaboxadol binding at low ligand concentrations in other brain regions, consistent with the low levels of α_4_ and δ-GABA_A_R subunit protein[32].

### Gaboxadol activates a tonic change in whole-cell baseline current blocked by gabazine

**Figure 2:**
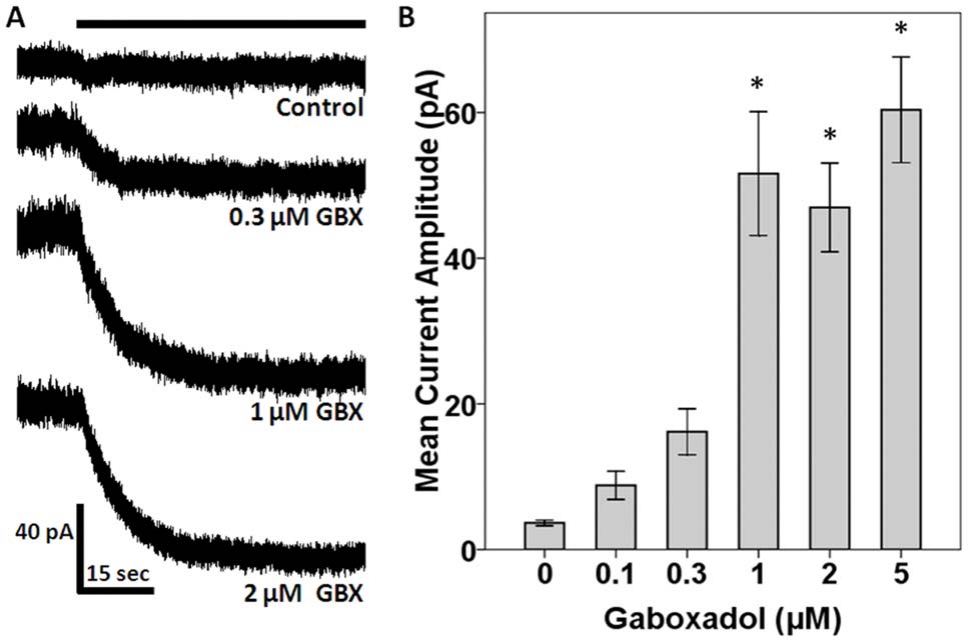
GABA_A_R Mediated Tonic Inhibition in MGB Neurons: **A**) Representative traces of gaboxadol-induced tonic Cl^−^ currents (outward) revealed by gabazine block, resulting in an inward shift in baseline current for MGB neurons held at −10 mV. The solid black line above the first trace represents the continuous focal application of (50 µM) gabazine for all traces. **B**) Bar graph of tonic current amplitude changes revealed by focal application of gabazine in the presence of increasing concentrations of GABA_A_R agonist, gaboxadol (GBX), applied to the ACSF. Current amplitudes are represented on the y-axis with the concentration of gaboxadol on the x-axis. (*p<0.005 when compared to Control using Dunnett's post-hoc analysis, data underwent first-order Winsorization; n = control: 8; 0.1 µM: 4; 0.3 µM: 6; 1 µM: 6; 2 µM: 6; 5 µM: 6).

*In vitro* whole cell voltage-clamp recordings from visually identified neurons in the MGv were conducted in control ACSF or in ACSF containing gaboxadol (0.1, 0.3, 1, 2 or 5 µM). Under these experimental conditions, activation of extrasynaptic GABA_A_Rs caused a shift in baseline current (in voltage-clamp) or potential (in current-clamp), suggesting the functional expression of α_4_δ-GABA_A_Rs in recorded neurons. For each condition, focal application of gabazine (50 µM), a selective GABA_A_R antagonist, was used to block all GABA_A_R-mediated currents, revealing the presence of constitutive (control) and/or gaboxadol elicited tonic currents. The membrane potential was clamped at −10 mV, therefore GABA_A_R-mediated currents were detected as outward shifts. Gabazine blockade caused a decrease in tonic outward current, represented as an inward shift in baseline current (Figure 2A). The Cl^−^ equilibrium potential was estimated to be near −60 mV. The difference between the holding current before and during focal gabazine application (ΔI) was defined as the amplitude of the tonic current in response to activation of α_4_δ-GABA_A_Rs. The value of ΔI increased as a function of gaboxadol concentration, however even in the absence of gaboxadol, a small tonic current was detected supporting the presence of constitutive activation of extrasynaptic α_4_δ-GABA_A_Rs in MGB neurons (Figure 2B).

In an effort to address the disparity in subject age between the two sets of experiments, MGB neurons from adult (6-7-month-old) FBN rats were examined. In 6-month-old neurons ΔI for 1 µM gaboxadol was 86.4±22.8 pA (n = 3; mean±SEM). Analyzed as current density to account for any developmental morphological changes, ΔI for 1 µM gaboxadol was significantly greater in 6-month-old MGv neurons (0.91±0.05 pA/pF; n = 3) in comparison to 22-30-day-old MGv neurons (0.50±0.09; n = 6) (t-test; p = 0.024).

## Discussion

These findings strongly support the presence of functional α_4_δ-GABA_A_Rs in MGB neurons. Receptor-binding autoradiorgraphy at low ligand concentrations of the subunit selective GABA_A_ superagonsit gaboxadol [33] show evidence of α_4_δ-GABA_A_Rs expression on auditory thalamocortical cell membranes while whole cell recordings from brain slices provide evidence for functional likely extrasynaptic α_4_δ-GABA_A_Rs mediating tonic inhibition. Tonic GABA_A_R mediated inhibition was recorded from neurons in 22-30-day-old and 6-7-month-old animals for consistency with [^3^H]gaboxadol binding results. These recordings suggest a qualitative similarity between 22-30-day-old and adult MGB neurons, but find a developmental increase in the amplitude of the tonic current. This increase in current amplitude likely reflects the developmental increase in δ-subunit containing GABA_A_R expression seen in the cerebellum and thalamus [29].

**Figure 3:**
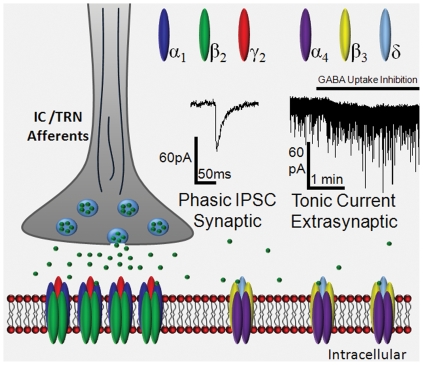
Summary Illustration of GABA_A_Rs on MGB Neurons: Note receptor location relative to the presynaptic GABAergic terminal (IC or TRN) with classic α_1_γ-subunit containing GABA_A_Rs located within the synapse and α_4_δ-subunit containing GABA_A_Rs outside of the synapse. The concentration of GABA to which each receptor type is typically exposed and the nature of the current mediated by each subtype of receptor is also depicted. For sample traces, the internal solution used here is CsCl-based (140 mM) and the membrane potential is clamped at −60 mV. As a result, GABA_A_R currents are inward and blocked by gabazine. The phasic response (left trace) is expanded from within the trace of the tonic response (right trace). The inward shift in baseline current is induced by upregulating extracellular GABA through inhibition of GABA uptake via the application of neuronal and glial GABA transporters with NNC-711 and SNAP 5114, respectively (solid line, right trace). These sample recordings were obtained from an MGB neuron of a 7-month-old FBN rat.

GABAergic inputs onto MGB neurons from IC and mostly from TRN are likely to activate tonic GABA_A_R currents through feedforward or feedback inhibition (Figure 3) [1], [7], [9], [10], [11], [12], [13], [16]. As α_4_δ-GABA_A_Rs have been shown to regulate neuronal excitability different from classical synaptic GABA_A_Rs in other sensory systems, they may prove to play a significant role in processing acoustic information. Enhanced inhibitory tone in auditory thalamic neurons may then serve to increase signal fidelity by decreasing “jitter” or noise level through hyperpolarization of the resting membrane potential [34]. A GABA-induced persistent hyperpolarization would lower the probability of excitatory input generated by intrinsic background noise to cause depolarizations great enough to reach action potential threshold. This damping/inhibition may be most important when coding temporally complex sounds like speech under severe/noisy listening conditions. However, work in understanding the role α_4_δ-GABA_A_Rs in stimulus coding in sensory thalamus is sparse.

The effect of α_4_δ-GABA_A_R activation on cellular excitability has been examined in the studies described above but understanding the role of this receptor subtype in terms of neurotransmission per se has received less attention. For example, hyperpolarization via α_4_δ-GABA_A_R mediated tonic inhibition has been shown to be involved in the transition from a tonic to burst response mode in thalamic neurons through the hyperpolarized potential's interaction with T-type Ca^2+^ channels [22]. It follows that this receptor subtype may participate in the generation of thalamic oscillations. Exactly what this means in an intact system has not been determined but dysfunctional tonic inhibition in the MGB may be associated with tinnitus [35]. An additional hypothesis for a functional role of α_4_δ-GABA_A_Rs is in mediating novelty detection through stimulus specific adaptation [1], [3]. Increased ambient GABA levels from TRN inhibitory afferents to MGB could contribute to decreased stimulus evoked firing rates through the activation of tonic GABA_A_R mediated hyperpolarizing currents. This type of regulation of firing rates in response to repetitive acoustic stimuli in the MGB may be similar to what is observed in stimulus specific adaptation in the IC and AC [36], [37], [38]. Recently, the TRN, a major part of the network responsible for generating rhythmic thalamic oscillations, was shown to play an integral role in the detection of novel stimuli by MGB neurons [1]. In conclusion, while the physiological role of tonic inhibition and how exactly it influences neurotransmission is still under investigation, candidate roles for α_4_δ-GABA_A_Rs in the auditory system include, but are not limited to, the regulation of general cellular excitability, enhancement of temporal coding fidelity and novel stimuli detection. Future studies will focus on examining the role of α_4_δ-GABA_A_Rs in the auditory thalamus, providing insight into how tonic inhibition may contribute to the extraction and/or processing of meaningful sounds. Finally, this GABA_A_ receptor subtype could provide a unique target for new therapeutic agents to treat tinnitus or improve speech processing in age-related hearing loss, two maladies thought to involve the selective down-regulation of inhibition as a function of partial peripheral deafferentation.
